# Emerging Functions of Actins and Actin Binding Proteins in Trypanosomatids

**DOI:** 10.3389/fcell.2020.587685

**Published:** 2020-10-09

**Authors:** Chhitar M. Gupta, Bindu Ambaru, Rani Bajaj

**Affiliations:** ^1^Institute of Bioinformatics and Applied Biotechnology, Bengaluru, India; ^2^Manipal Academy of Higher Education, Manipal, India

**Keywords:** tryanosomatids, actin, actin binding proteins, intracellular distribution, structure, functions

## Abstract

Actin is the major protein constituent of the cytoskeleton that performs wide range of cellular functions. It exists in monomeric and filamentous forms, dynamics of which is regulated by a large repertoire of actin binding proteins. However, not much was known about existence of these proteins in trypanosomatids, till the genome sequence data of three important organisms of this class, viz. *Trypanosoma brucei*, *Trypanosoma cruzi and Leishmania major*, became available. Here, we have reviewed most of the findings reported to date on the intracellular distribution, structure and functions of these proteins and based on them, we have hypothesized some of their functions. The major findings are as follows: (1) All the three organisms encode at least a set of ten actin binding proteins (profilin, twinfilin, ADF/cofilin, CAP/srv2, CAPz, coronin, two myosins, two formins) and one isoform of actin, except that *T. cruzi* encodes for three formins and several myosins along with four actins. (2) Actin 1 and a few actin binding proteins (ADF/cofilin, profilin, twinfilin, coronin and myosin13 in *L. donovani*; ADF/cofilin, profilin and myosin1 in *T. brucei*; profilin and myosin-F in *T.cruzi*) have been identified and characterized. (3) In all the three organisms, actin cytoskeleton has been shown to regulate endocytosis and intracellular trafficking. (4) *Leishmania* actin1 has been the most characterized protein among trypanosomatid actins. (5) This protein is localized to the cytoplasm as well as in the flagellum, nucleus and kinetoplast, and *in vitro*, it binds to DNA and displays scDNA relaxing and kDNA nicking activities. (6) The pure protein prefers to form bundles instead of thin filaments, and does not bind DNase1 or phalloidin. (7) Myosin13, myosin1 and myosin-F regulate endocytosis and intracellular trafficking, respectively, in *Leishmania*, *T. brucei* and *T. cruzi*. (8) Actin-dependent myosin13 motor is involved in dynamics and assembly of *Leishmania* flagellum. (9) *Leishmania* twinfilin localizes mostly to the nucleolus and coordinates karyokinesis by effecting splindle elongation and DNA synthesis. (10) *Leishmania* coronin binds and promotes actin filament formation and exists in tetrameric form rather than trimeric form, like other coronins. (11) Trypanosomatid profilins are essential for survival of all the three parasites.

## Introduction

Eukaryotic cell cytoskeleton is a dynamic structure comprised of three components, viz. microfilaments, microtubules and intermediate filaments. Actin is the major protein constituent of the microfilaments, which regulates a variety of cell functions, such as motility (Theriot and Mitchison, 1991; Pollard and Borisy, 2003), cell division (Pollard, 2008), endocytosis, intracellular trafficking (Girao et al., 2008; Khaitlina, 2014), chromatin remodeling, DNA repair and regulation of transcription (Bettinger et al., 2004; Miralles and Visa, 2006; Percipalle and Visa, 2006; Chen and Shen, 2007; Hurst et al., 2019). The dynamics (assembly and disassembly) of actin microfilaments is regulated by a large array of actin binding proteins (dos Remedios et al., 2003; Pollard, 2016), activities of which in turn are controlled by specific signaling pathways (Mackay and Hall, 1998).

Trypanosomatids are protozoan parasites that infect invertebrate hosts. But some of them also infect humans and animals, where the invertebrate host serves as the vector that facilitates their transmission. As microtubules constitute most of the cytoskeleton network in trypanosomatids and there was no convincing evidence until the year 2004 on the role of actin and its network proteins in these organisms, it was believed that actin cytoskeleton perhaps has no major role in their cellular activities (Gull, 1999). Nevertheless, the genome analysis data of trypanosomatids, especially *Trypanosoma brucei*, *Trypanosoma cruzi* and *Leishmania major*, revealed that their genomes contained genes that putatively encode for actin and several actin binding proteins (Berriman et al., 2005; El-Sayed et al., 2005; Ivens et al., 2005), some of which have now been characterized and in few instances, their functions have been unraveled. Here, we have critically reviewed all the findings that have been reported to date on actin and actin binding proteins in trypanosomatids, and based on the available knowledge, we have hypothesized their potential roles in cellular activities of these organisms.

## Brief Overview of Conventional Actins

Actin is an ancient and highly conserved protein present in all eukaryotic cells, which shares a high amino acid sequence identity among widely diverse groups of eukaryotes, for example *Caenorhabditis elegans* and *Homo sapiens*, sharing about 90% sequence identity in their actins. Even in bacterial cells, the presence of distant homologues of actin, such as MreB, FtsA and ParM, have been identified (Graumann, 2007; Pogliano, 2008). While MreB protein functions in regulating the bacterial cell shape and cell wall synthesis, FtsA and ParM participate, respectively, in the bacterial cell division and plasmid segregation (Graumann, 2007; Pogliano, 2008). Additionally, more than 30 genes encoding for actin-like proteins (ALPs) have been characterized in bacteria which are mainly present on plasmids and bacteriophage genomes (Derman et al., 2009). Further, lineages of archaea which are closely related to eukaryotes have been identified to encode for close homologues of eukaryotic actin (Braun et al., 2015; Spang et al., 2015; Zaremba-Niedzwiedzka et al., 2017).

The ancient actin gene during early evolution crossed over several times with genes of other species, giving rise to genes for actin-related proteins, called “Arps.” These genes further diversified into several families exhibiting differing functions, when the common progenitor of animals, fungi, and amoebas diverged from the large clade of organisms, including algae, plants, and a variety of other single-celled organisms (Muller et al., 2005). Arps share 17–52% amino acid sequence identity with actin and depending on their divergence from actin, these proteins have been numbered from Arp1 to Arp11, wherein Arp1 has the maximum and Arp 11 the minimum closeness to actin (Muller et al., 2005). These proteins have been further divided, depending on their presence in the cells, into two groups– cytoplasmic and nuclear Arps. Whereas Arp 1-3, Arp10 and Arp11 have been classified into cytoplasmic group of Arps, Arp4-Arp9 constitute the nuclear group (Muller et al., 2005), which participate in chromatin remodeling and other related nuclear functions (Oma and Harata, 2011; Maruyama et al., 2012).

Actin is a 375 amino acids long globular polypeptide that primarily exists in monomeric (G-actin) and filamentous (F-actin) forms. In cells, monomeric actin is almost exclusively found in the ATP-bound state, whereas in filamentous form, it largely exists in the ADP-bound form. Actin polymerization comprises three steps, which in the first step involves a slow association (called “lag phase”) of two actin monomers to form a dimer that has high tendency to revert back to monomers rather than to assemble further. This is followed by the formation of a stable trimer that serves as the nucleus for further polymerization, and finally the “elongation phase,” where filament assembly takes place faster due to rapid association of ATP-bound G-actin to the polar (or barbed) end of growing filament (dos Remedios et al., 2003). The polymerization process is promoted by the presence of divalent cations under physiological conditions. The ATP bound to actin in filaments is hydrolyzed into ADP and phosphate, and as filament matures, the phosphate is released with concomitant dissociation of ADP-actin from the pointed end. The ADP bound G-actin released from the filament, after undergoing exchange of ADP for ATP, can then undergo fresh round of polymerization. In a steady state, a dynamic equilibrium is reached where the length of the actin filaments remains constant, with actin monomers continually associating to and dissociating from the ends. This process is referred to as “actin treadmilling.”

**FIGURE 1:**
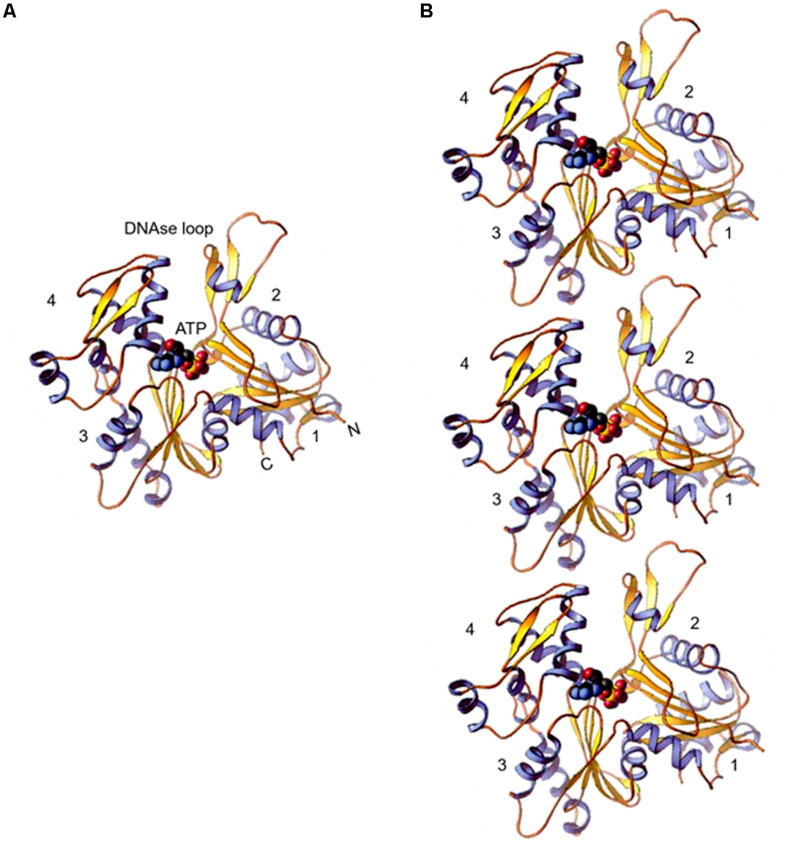
**(A)** Ribbon diagram of the actin molecule with space filling ATP (protein data bank [PDB]: 1ATN). N, amino terminus; C, carboxyl terminus. Numbers 1, 2, 3, and 4 label the four subdomains (re-printed from Pollard et al. (2016) with copyright permission from Elsevier Publishers). **(B)** Model of actin protofilaments derived from linear polymers along a single strand of F-actin.

The inherent tendency of actin to polymerize into filaments hindered for decades the growth of actin crystals, suitable for determining its 3-d structure. Eventually, in the 1990s high resolution 3-d structure was resolved separately for co-crystals of actin with DNase I (Kabsch et al., 1990) and profilin (Schutt et al., 1993), and also for free G (monomer)-actin (Chik et al., 1996). Afterward, several actin structures have been reported (Dominguez and Holmes, 2011) and in all, the conformation of actin monomer remained fundamentally the same. The actin polypeptide folds into one large and one small domain. These domains further have two sub-domains each. While the small domain comprises subdomain 1 and subdomain 2, the large domain comprises subdomain 3 and subdomain 4. Between these sub-domains two clefts are formed – “nucleotide binding cleft,” that binds to ATP or ADP with a divalent cation and the other one is the “target binding cleft,” which is hydrophobic and defines the region where most actin binding proteins (ABPs) and small molecules interact with actin and also where actin subunits make contacts in the filament (Figure 1). Depending on the state of bound nucleotide in the “nucleotide binding cleft,” structure of actin monomer changes, which in turn modulates the binding affinities of “target binding cleft” for ABPs and also alters the strength of actin monomer interaction in the filament. Further, the DNase I binding site is formed from amino acid residues (aa) 39–46 and 60–64 of subdomain 2 and aa 202–204 and 207 of subdomain 4 of which aa 40–50 of subdomain 2 are highly disordered, that form the DNase I binding loop.

Assembly of actin filaments involves association of subdomains 2 and 4 of one G-actin molecule with subdomains 1 and 3 of the other molecule. A part of amino acid sequences that are contributed by subdomain 2 in this process constitute the DNase I binding site. A loop of eleven aa residues (aa 40–50 of subdomain 2) that also include a four aa residues hydrophobic plug then stabilizes the filament. This loop inserts into the hydrophobic pocket formed by subdomains 2 and 3 of adjacent monomers on the opposing strands (Figure 1). Based on the X-ray diffraction pattern of oriented F-actin gels, Holmes et al. (1990) proposed the first structural model of actin filament, which was further modified by Oda et al. (2009). The modified model illustrated that actin monomers are arranged in a two-start filament of 7–10 nm thickness having a half pitch of 37 nm and a rise of 2.75 nm per monomer. A large number of proteins associate with and effect the functions of actin by remodeling its network in cells (dos Remedios et al., 2003; Pollard et al., 2016; Merino et al., 2020). These proteins are mostly conserved in a wide variety of eukaryotes. Some members belonging to these proteins by virtue of their actin monomer sequestering activity affect availability of the polymerizable pool of free actin monomers, while there are others that control filament formation and stability through their nucleating, elongating, depolymerizing, severing, capping, crosslinking and bundling activities (Winder and Ayscough, 2005).

The main features that define conventional (or canonical) actins are based on their following properties: (1) they form long and stable filaments having width between 7 and 10 nm in the presence of a divalent cation as Mg^+2^, with or without ATP; (2) they bind DNase I and inhibit its activity; (3) their filaments are stabilized by phallotoxins and destabilized by cytochalasins or latrunculins (Reisler, 1993; Wakatsuki et al., 2001); and (4) their filament dynamics is regulated by a set of about 20 core ABPs that include actin depolymerizing factor (ADF)/cofilins, twinfilin, profilin, gelsolin, CAP/Srv2, formin, Arp2/3 complex, β-thymosin, troponin, filamin, fimbrin, villin, actinin, plastin, spectrin and CapZ. However, lower eukaryotic organisms such as *Plasmodium*, *Toxoplasma*, *Trypanosoma*, *Leishmania*, *Giardia*, *Amoebae and Ciliate* group of protozoans contain actins which display highly unusual characteristics (Villalobo et al., 2001; Gupta et al., 2015). While some of these organisms express actins and ABPs that exhibit unusual biochemical and functional characteristics, there are others, such as *Giardia lamblia*, which express single copy of highly divergent actin (Drouin et al., 1995), and their genome lacks genes that encode the core ABPs, which are essentially required to regulate actin dynamics in higher eukaryotic organisms (Morrison et al., 2007; Pollard, 2016). Yet these organisms utilize their actin similar to other eukaryotic actins in their all vital cellular functions, such as morphogenesis, intracellular trafficking and cytokinesis (Paredez et al., 2011, 2014).

## Classification of Protozoan Organisms

**FIGURE 2:**
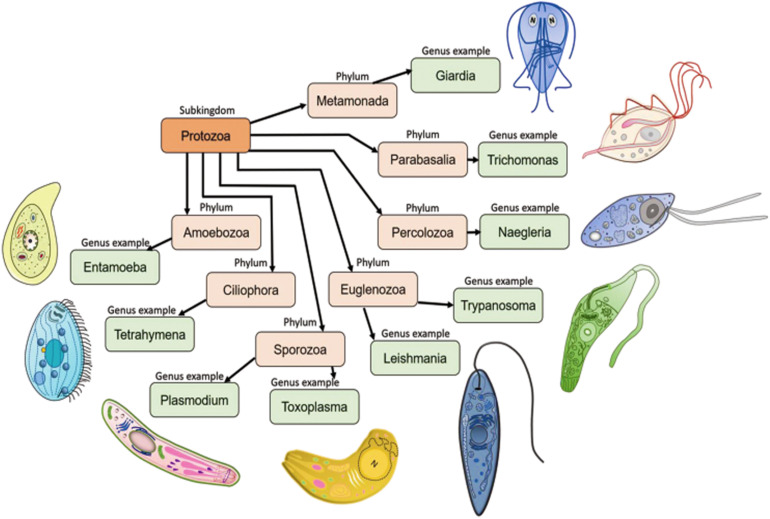
Seven important phyla of subkingdom protozoa with schematic representations. Metamonada (intestinal flagellates, e.g., *Giardia*); Parabasalia (intestinal and related flagellates, e.g., *Trichomonas*); Percolozoa (flagellated amoebae, e.g., *Naegleria*); Euglenozoa (kinetoplastid flagellates, e.g., *Trypanosoma*, *Leishmania*); Amoebozoa (amoebae, e.g., *Entamoeba*); Sporozoa (sporozoans, e.g., *Toxoplasma*, *Plasmodium*) and Ciliophora (ciliates, e.g., *Tetrahymena*). Schematic images of the protozoan parasites.

Protozoans are single-celled microscopic eukaryotic organisms of a group of phyla of the kingdom *Protista*. In the widely used 1980 classification based on locomotion (Levine et al., 1980), the protozoan subkingdom was classified into seven phyla which included the *Sarcomastigophor*a (combination of *Mastigophora* and *Sarcodina*), *Apicomplexa*, *Microspora*, *Myxozoa* and *Ciliophora*. The most recent classifications recognized 13 phyla, of which seven contain important parasites (Figure 2): *Metamonada* (intestinal flagellates, e.g., *Giardia*); *Parabasalia* (intestinal and related flagellates, e.g., *Trichomonas*); *Percolozoa* (flagellated amoebae, e.g., *Naegleria*); *Euglenozoa* (kinetoplastid flagellates, e.g., *Trypanosoma*, *Leishmania*); *Amoebozoa* (amoebae, e.g., *Entamoeba*); *Sporozoa* (sporozoans, e.g., *Toxoplasma*, *Plasmodium*) and *Ciliophora* (ciliates, e.g., *Tetrahymena*) (Levine et al., 1980; Cavalier-Smith, 2002; Cox, 2002). Further, kinetoplastids comprise five orders: *Trypanosomatida*, *Eubodonida*, *Parabodonida*, *Neobodonida* and *Prokinetoplastida* (Moreira et al., 2004). Within kinetoplastids, the most studied family is the *Trypanosomatidae*, which comprises mainly of monoxenous parasite species that infect invertebrates (*Leptomonas*) and of dixenous species that can be pathogenic to plants, animals and/or humans (*Phytomonas*, *Trypanosoma* and *Leishmania*) (Kaufer et al., 2017). *Trypanosomatidae* got much of their fame because of the two genera, *Trypanosoma* and *Leishmania*, which cause African sleeping sickness (*Trypanosoma brucei*), Chagas disease (*Trypanosoma cruzi*), visceral Leishmaniasis (*Leishmania donovani*, *Leishmania infantum*, *Leishmania chagasi*), cutaneous Leishmaniasis (*L. major*, *L. panamensis*, *L. tropicana*) and mucocutaneous Leishmaniasis (*L. braziliensis*). In the following sections, we will revisit all the findings reported to date on characterization, intracellular localization and functions of trypanosomatid actins and actin binding proteins, and based on the available knowledge, we will attempt to stipulate their functions in these organisms.

## Trypanosomatid Actins and Actin Binding Proteins

### Trypanosomatid Actins

**FIGURE 3:**
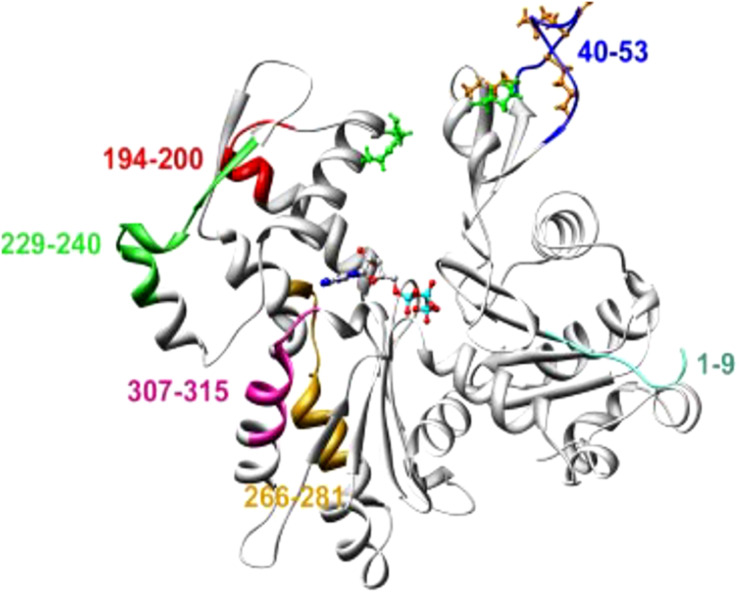
An average molecular dynamics simulated homology model of LdAct showing colored stretches of diverged amino acid residues (aa 1–9 of subdomain 1, aa 40–53 of subdomain 2, aa 266–281 and aa 307–315 of subdomain 3, aa 194–200 and 229–240 of subdomain 4), brown ball and stick residues in the DNase-I binding loop are the diverged replacements in LdAct that are known to make strong interactions with DNase-I in the actin-DNase-I complex crystal structure, whereas green ball and stick residues are conserved amino acid residues that are known to make weak interactions with DNase-I (taken from Kapoor et al., 2008 with permission).

Trypanosomatid actins possessed approximately 70% aa identity to human or yeast actin (Gupta et al., 2015). The major differences in the aa sequence were confined to the aa1–9, aa 40–53, aa194–200, aa 229–240, aa 266–281 and aa307–315, most of which were located on the surface of yeast or mammalian actin (Figure 3). Domain-wise analysis revealed that subdomain 2 (aa33– 69), subdomain 3 (aa145–180 and aa270–337) and subdomain 4 (aa181–269) have higher divergence (30–40%) from the corresponding subdomains of human actin, compared with subdomain 1 of *Leishmania* actin (aa1–32, 70–144 and aa338–375). These diverged amino acid sequences partly included the sites that are engaged in actin self-association and DNase I binding.

The presence of actin gene in trypanosomatid organisms was established about 30 years ago (Ben Amar et al., 1988; de Arruda and Matsudaira, 1994), but all earlier attempts to isolate and characterize actin protein, using conventional methods, met with failure, primarily due to lack of these proteins to bind DNase I (Mortara, 1989). Also, fluorescently labeled phalloidin staining failed to identify the presence of filament-like structures in trypanosomatids, especially *Leishmania* cells (Mortara, 1989). Further, by using electron microscopy, no filament-like structures corresponding to mammalian actin filaments (5–7 nm diameter) could be seen in these cells (Gull, 1999), suggesting that actin may not have any major role in cellular functions of trypanosomatids (Gull, 1999). However, genomic analysis of three major pathogenic organisms of trypanosomatid family, viz. *T. brucei*, *T. cruzi* and *Leishmania spp.*, identified genes that putatively encode at least for one copy of conventional actin (Act1), similar to other eukaryotic actins, and numerous actin-like, actin-related and actin binding proteins (Tables 1 and 2), revealing the presence of a dynamic actin network in trypanosomatids. Compared with *T. brucei* and *L. major*, *T. cruzi* appears to have more complex actin cytoskeleton, as its genome encodes for multiple copies of actin and an expanded set of actin binding proteins (Berriman et al., 2005). *T. cruzi* has as many as four actin genes of which TcAct1 and TcAct2 have been characterized (De Melo et al., 2008; Vizcaíno-Castillo et al., 2019). However, actin 2 and 3 are absent in *T. brucei*, *L. donovani*, *L. major* and *L. braziliensis*. The fourth actin is encoded by *T. cruzi* and *L. major*, but not by *T. brucei* (Cevallos et al., 2011; Vizcaíno-Castillo et al., 2019). This actin isoform is also present in *L. donovani* and *L. braziliensis*, but its annotation has been given as actin-like protein. Besides actins, a variable number of actin-like and actin related proteins are also encoded by the trypanosamatid genomes (recently reviewed in Vizcaíno-Castillo et al., 2020).

**TABLE 1:** Presence of actin and actin-like proteins in five main disease causing trypanosomatids.

	*Trypanosoma cruzi*	*Trypanosoma brucei*	*Leishmania donovani*	*Leishmania major*	*Leishmania braziliensis*
	Chagas disease	Sleeping sickness	Visceral Leishmaniasis	Cutaneous Leishmaniasis	Mucocutaneous Leishmaniasis
Actin	TcAct1* (TcCLB.510571.30) (TcCLB.510127.79)	ActinA* (Tb927.9.8850) ActinB (Tb927.9.8880)	LdAct (LDBPK_041250)	LmjF.04.1230	LbrM.04.1250
Actin 2	TcAct2 (TcCLB.507129.10)	Absent	Absent	Absent	Absent
Actin, putative (actin 3rd)	TcCLB.510945.30	Absent	Absent	Absent	Absent
Actin, putative (actin 4th)	TcCLB.503841.40	Absent	LdBPK_350810.1 (actin-like protein)	LmjF.35.0790 (actin-like protein)	LbrM.34.0780 (actin-like protein)
Actin -like protein 1	TcCLB.508277.330	Tb927.9.5440	LdBPK_151350.1	LmjF.15.1330	LbrM.15.1280
Actin -like protein 2	TcCLB.506405.30	Tb927.4.980	LdBPK_343560.1	LmjF.34.3760	LbrM.20.3360
Actin -like protein 3	TcCLB.506733.50	Tb927.11.3880	LdBPK_130840.1	LmjF.13.0950^#^	LbrM.13.0760
Actin -like protein 4	TcCLB.510719.110	Tb927.11.10110	LdBPK_363470.1	LmjF.36.3310	LbrM.35.3540
Actin -like protein 5	TcCLB.506695.10	Tb927.3.3020	LdBPK_292850.1	LmjF.29.2740	LbrM.29.2800
Member of ARP6 family (Actin like protein)	TcCLB.508951.29	Tb927.10.2000	LdBPK_210290.1	LmjF.21.0230	LbrM.21.0300

The data was downloaded from the TriTrypDB version 48 database (www.tritrypdb.org). IDs of strains used: *T. cruzi* CL Brener Esmeraldo-like; *T. brucei brucei* TREU927*; L. donovani* BPK282A1*; L. major* strain Friedlin*; L. braziliensis* MHOM/BR/75/M2904. *This actin locus contains two copies of the gene on one allele and one copy on the second allele (Ben Amar et al., 1988; Cevallos et al., 2003). ^#^This has been referred as actin-like protein 3 in Cevallos et al. (2011) and as ARP1 in Singh et al. (2014).

**TABLE 2:** Presence of actin binding proteins in five main diseases causing trypanosomatids, compared to higher eukaryotes.

	Higher eukaryotes	*Trypanosoma cruzi*	*Trypanosoma brucei*	*Leishmania donovani*	*Leishmania major*	*Leishmania braziliensis*
Disease caused	**NA**	Chagas disease	Sleeping sickness	Visceral Leishmaniasis	Cutaneous Leishmaniasis	Mucocutaneous Leishmaniasis
Actin monomer binding	Profilin	TcCLB.510911.10	Tb927.11.13780	LdBPK_320550.1	LmjF32.0520	LbrM.32.0570
	Thymosin B4	Absent	Absent	Absent	Absent	Absent
	ADF/Cofilin	TcCLB.510145.20	Tb927.3.5180	LdBPK_290520.1	LmjF29.0510	LbrM.29.0450
	Gelsolin	Absent	Absent	Absent	Absent	Absent
	Twinfilin	TcCLB.506559.300	Tb927.4.2350	LdBPK_342060.1	LmjF.34.2290	LbrM.20.1790
	CAP/Srv2	TcCLB.504137.80	Tb927.10.9250	LdBPK_365830.1	LmjF36.5590	LbrM.35.5860
Filament binding	Myosin	TcCLB.511527.70 (myosin 13) TcCLB.507739.110 (1B heavy chain) TcCLB.504867.120 MyoA TcCLB.506779.190 MyoB TcCLB.504103.30 MyoC TcCLB.503905.10 MyoD TcCLB.503905.10 MyoE TcCLB.507445.50 MyoF TcCLB.507093.210 MyoG	Tb927.11.16310 (Unconventional myosin) Tb927.4.3380 (1B heavy chain) Tb927.9.1340 (myosin like protein 2) Tb927.11.330 (myosin like protein 1)	LdBPK_324020.1 (myosin XXI) LdBPK_341070.1 (1B heavy chain)	LmjF.32.3870 (myosin XXI) LmjF.34.1000 (1B heavy chain)	LbrM.32.4110 (myosin XXI) LbrM.20.0970 (1B heavy chain)
	Coronin	TcCLB.510515.100	Tb927.8.3100	LdBPK_231400.1	LmjF.23.1165	LbrM.23.1260
	CAPz	TcCLB.506181.90 TcCLB.506363.60	Absent	Absent	Absent	Absent
Nucleating	Arp2/3 complex (7 subunits) Arp2	TcCLB.511361.40	Tb927.10.15800	LdBPK_191190.1	LmjF19.1200	LbrM.19.1370
	Arp3	TcCLB.508277.260	Tb927.9.5350	LdBPK_151410.1	LmjF.15.1360	LbrM.15.1360
	ARPC1	TcCLB.504215.40	Tb927.10.13190	LdBPK_180920.1	LmjF.18.0920	LbrM.18.0980
	ARPC2	TcCLB.506865.10	Tb927.8.4410	Absent	Absent	Absent
	ARPC3	TcCLB.510963.70	Tb927.10.4540	Absent	Absent	Absent
	ARPC4	TcCLB.509127.104	Tb927.2.2900	LdBPK_020570.1	LmjF.02.0600	LbrM.02.0580
	ARPC5	TcCLB.442297.10	Tb927.10.10600	LdBPK_050290.1	LmjF.05.0285	LbrM.05.0280
	ARPC-like	Absent	Absent	LdBPK_101080.1	LmjF.10.1000	LbrM.10.1100
	Formin	TcCLB.511313.30 TcCLB.506203.80 TcCLB.511393.30	Tb927.5.2300 Tb927.11.5740	LdBPK_171040.1 LdBPK_241130.1	LmjF24.1110 LmjF17.0930	LbrM.17.0950 LbrM.24.1120
Crosslinking proteins	Fimbrin, villin, α- actinin, plastin, spectrin, filamin	Absent	Absent	Absent	Absent	Absent

*The data was downloaded from the TriTrypDB version 48 database (www.tritrypdb.org). IDs of strains used: *T. cruzi* CL Brener Esmeraldo-like; *T. brucei brucei* TREU927; *L. donovani* BPK282A1*; L. major* strain Friedlin*; L. braziliensis* MHOM/BR/75/M2904.*

The presence and intracellular distribution of Act1 in all the trypanosomatids studied so far have been analyzed by employing polyclonal antibodies against recombinant version of Act1, as a probe. This technique enabled to identify differences in the subcellular distribution of Act1 not only in different species, but also in the different developmental stages. TbAct1 was equally expressed in both the blood stream and procyclic stages of *T. brucei*, but in blood stream stage, it was more enriched at the posterior end and colocalized with the endocytic pathway (García-Salcedo et al., 2004). However, in procyclic form, it was distributed throughout the cytoplasm (García-Salcedo et al., 2004). Unlike *T. brucei*, there have been numerous contradictory reports on the intracellular distribution in the various forms of *T. cruzi*. Presence of TcAct1 in *T. cruzi* epimastigotes was first reported by de Souza et al. (1983), using polyclonal anti-rabbit muscle actin antisera, wherein they claimed that this protein was sparsely distributed throughout the cell body and the paraxial structure of the flagellum. Subsequent studies using polyclonal antibodies against the conserved N-terminal region of TcAct1 showed that TcAct1 was distributed in patch-like structures throughout the cytoplasm in epimastigote, amastigote and bloodstream trypomastigote forms of *T. cruzi* (De Melo et al., 2008). This distribution was further confirmed using polyclonal anti-recombinant TcAct1 antibodies (Cevallos et al., 2011). Further analysis revealed that these antibodies recognized a single band in one-dimensional electrophoresis, but in two-dimensional electrophoresis, it identified five isoforms of TcAct1 in all stages of the parasite development (Cevallos et al., 2011). Immunofluorescence analysis, using anti-recombinant TcAct1 antibodies, showed that TcAct1 was faintly distributed throughout the cell with intense staining at the base of the flagellum near the flagellar pocket area and along the flagellum in epimastigotes (Cevallos et al., 2011). In trypomastigotes, TcAct1 was uniformly distributed with a low level of staining (Cevallos et al., 2011), and in the cell-derived amastigotes, a heterogeneous TcAct1 localization with sometimes no apparent expression was observed (Cevallos et al., 2011). Similar protein expression levels and intracellular TcAct1 distributions in epimastigotes, amastigotes and metacyclic trypomastigotes were also observed by using mouse polyclonal anti-recombinant TcAct1 antibodies (Souza et al., 2013). In addition to TcAct1, TcAct2 has also been characterized. This protein was expressed throughout the life cycle of *T. cruzi* with several variants (Vizcaíno-Castillo et al., 2019). In all stages, TcAct2 did not co-localize withTcAct1, and had a diffused distribution throughout the cell body and in the flagellum, with a fine granular pattern (Vizcaíno-Castillo et al., 2019). Further, detergent fractionation of epimastigotes revealed that TcAct2 was a cytoplasmic rather than a cytoskeletal protein (Vizcaíno-Castillo et al., 2019). Because of differences in cellular localization of TcAct1 and TcAct2, it may be envisaged that these proteins possibly have non-redundant functions in *T. cruzi* cells.

**FIGURE 4:**
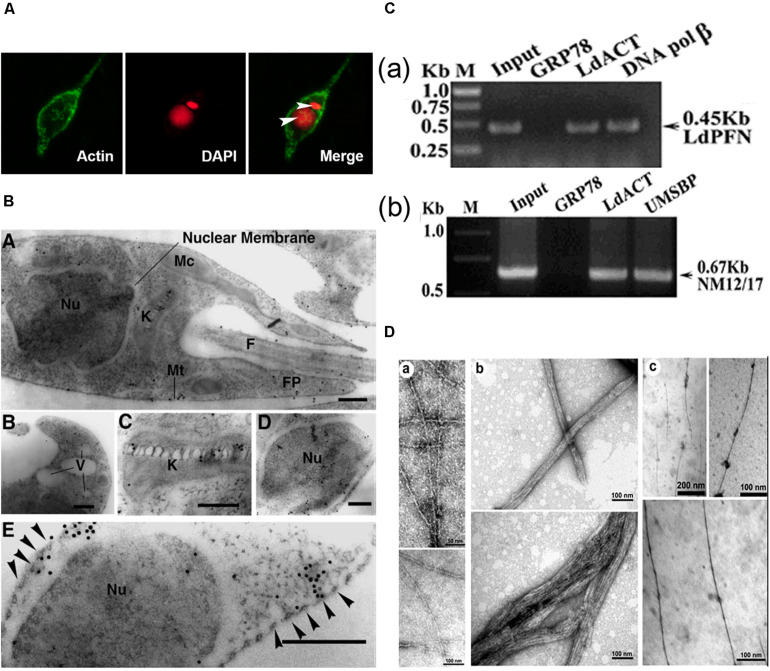
**(A)** Immunofluorescence micrograph of *Leishmania* promastigotes after treating them with 0. 5% NP-40 and staining with anti-LdAct antibodies and DAPI showing the presence of LdAct in the nucleus and kinetoplast and its association with nuclear DNA and kDNA (adapted from Sahasrabuddhe et al., 2004 with permission). **(B)** Electron micrographs of immunogold-labeled actin showing the presence (panel **a**) of LdAct in the nucleus (Nu), the kinetoplast (K), the flagellum (F), and the flagellar pocket (FP). In addition, the presence of LdAct on membranes of vacuoles (V) may also be noticed in panel **(b)**, and its associations with kDNA network, nuclear membrane and subpellicular microtubules may clearly be seen in panels **(c–e)**, respectively. The arrowheads in panel **(e)** mark the microtubules. Bar, 200 nm (Adapted from Sahasrabuddhe et al., 2004 with permission). **(C)** Chromatin Immuno-precipitation (ChIP) analysis using anti-LdAct antibodies showing the *in vivo* association of LdAct with chromatin **(a)** and kDNA network **(b)**. Panels **(a,b)** are the agarose gels of PCR products after ChIP assay. Lanes are marked on the top with their respective antibodies used in the ChIP assay and arrows indicated the genes amplified after pull down. An irrelevant, non-DNA associating antibody, GRP78, was used as a negative control, whereas antibodies against DNA polβ, and UMSBP (universal minicircle sequence-binding protein), were used as positive controls for nuclear DNA and kDNA respectively. LdPfn, *Leishmania* profilin; NM12/17, specific minicircle primers (this was originally published in Nucleic Acids Research, Kapoor et al., 2010^©^ Oxford University Press). **(D,a**) Negatively stained transmission electron micrograph of *in vitro* reconstituted rabbit muscle actin (RbAct) filaments in F-buffer (100 mM KCl, 2 mM MgCl_2_ and 2 mMATP; pH 8.0; 25°C) and **(b)** LdAct at 2 μM protein concentration, unlike RbAct, formed bundles rather thin filaments, under identical conditions. **(c)** LdAct forms very thin filaments at 0.2 μM G-LdAct concentration in F-buffer, pH7.0 at 25°C. RbAct under these conditions failed to form filaments (taken from Kapoor et al., 2008 with permission).

**FIGURE 5:**
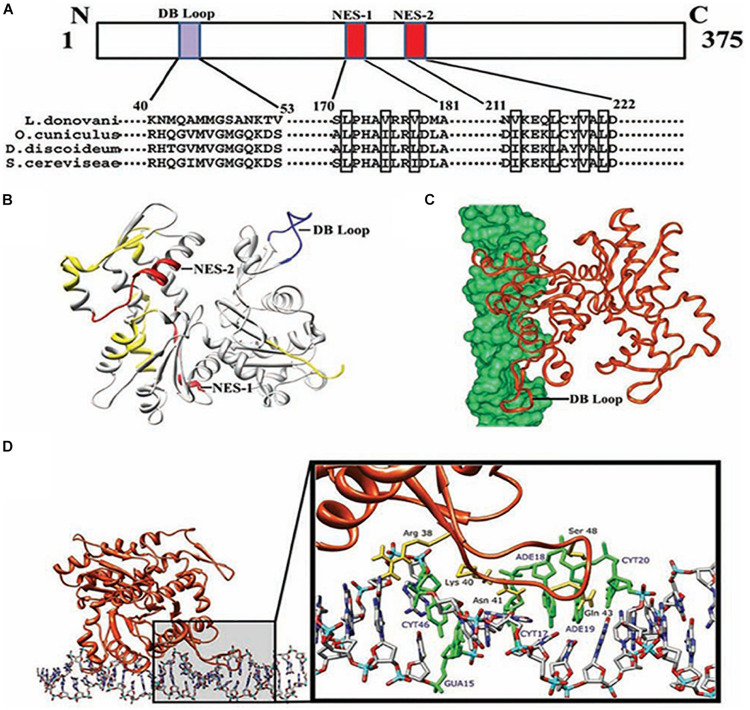
Computational docking of average simulated model of LdAct with DNA showing the interaction of the diverged DB-loop of LdAct with the major groove of DNA. **(A)** Sequence alignment of LdAct with other actins showing the presence of nuclear export signals (NES-1 and NES-2) in the LdAct aa sequence and the diverged DB-loop predicted to be involved in the DNA binding, by DP-Bind server. **(B)** Energy minimized average simulated model of LdAct showing positions of NES-1, NES-2 (red) and the diverged stretches of amino acid sequences (yellow) including the sequence that fall in DB loop (blue). **(C)** Docking of LdAct (orange) with DNA (green) using HADDOCK protocols. **(D)** Amino acid residues of the DB loop of LdAct (yellow) showing hydrogen bonding with the nucleotides (green) of DNA. DB, DNase I binding; NES, nuclear export signal (This was originally published in Nucleic Acids Research, Kapoor et al., 2010^©^ Oxford University Press).

**FIGURE 6:**
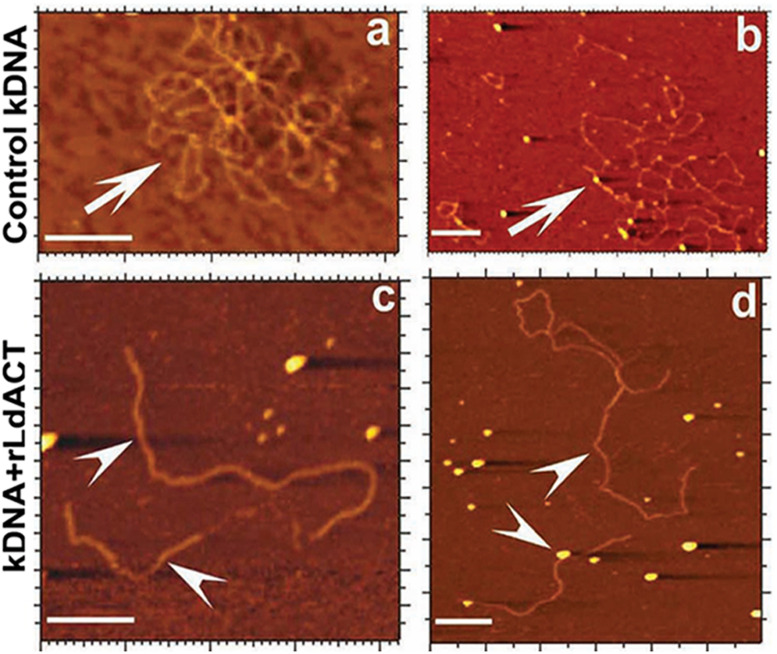
Atomic force micrographs of kDNA after its incubation in the presence and absence (control) of LdAct, showing decatenation of kDNA with LdAct. Panels **(a,b)** control kDNA, arrows indicate catenated kDNA. Panels **(c,d)** kDNA with rLdAct, arrowheads indicate decatenated nicked kDNA (scale bar: 500 nm) (This figure was originally published in Nucleic Acids Research, Kapoor et al., 2010^©^ Oxford University Press).

*L. donovani* Act1 (LdAct1) is the most characterized protein amongst all trypanosomatid actins (Sahasrabuddhe et al., 2004; Kapoor et al., 2008, 2010). This protein was abundantly expressed in both the promastigote and amastigote stages of *L. donovani* (Sahasrabuddhe et al., 2004). LdAct1 in *Leishmania* promastigotes was present as granules, patches, and filament-like structures throughout the cell body, including the flagellum, the nucleus and the kinetoplast (Sahasrabuddhe et al., 2004; Kapoor et al., 2008). These LdAct1 structures could not be stained with fluorescently labeled phalloidin nor could they be disrupted by treatment with cytochalasin D (Sahasrabuddhe et al., 2004). In the nucleus and the kinetoplast, LdAct1 was found to associate, respectively, with the chromatin and kDNA (Figures 4A–C). Besides this, recombinant LdAct1 (rLdAct1) polymerized *in vitro* to form bundles instead of thin filaments, only between pH 7.0 to pH 8.0 (Figure 4D), and its critical concentration of polymerization was 3–4 times lower than of rabbit muscle actin (Kapoor et al., 2008). In addition, it did not bind DNase I or phalloidin and during polymerization, it displayed significantly higher ATPase activity, compared with muscle actin (Kapoor et al., 2008). This apart, unlike any other eukaryotic actin, rLdAct bound to DNA primarily through electrostatic interactions involving its unique DNase-1-binding region and the DNA major groove (Figure 5) and relaxed negatively supercoiled DNA and nicked the kDNA, which converted kDNA minicircles into their open form (Figure 6), a unique property which no other eukaryotic actin has been found to have till date (Kapoor et al., 2010). The DNA nicking activity was largely confined to the DNase-1 binding loop, as treatment of LdAct1 with subtilisin, which was known to selectively cleave the DNase I binding loop without altering much the Act1 structure (Schwyter et al., 1989), significantly reduced its DNA nicking activity (Kapoor et al., 2010). Further, rLdAct1 inhibited the kDNA decatenation activity of bacterial type II topoisomerase (Kapoor et al., 2010), suggesting that LdAct1 may play an important role in remodeling of the chromatin and kDNA in trypanosomatids (Liu et al., 2005; Kapoor et al., 2010).

Trypanosomatid actins, similar to conventional actins, participate in the process of endocytosis. This process in *T. brucei* and *Leishmania* primarily occurs through the flagellar pocket (Morgan et al., 2002a), which is a well-defined structure formed from a lateral cell membrane depression that is continuous with the flagellar membrane. However, in *T. cruzi*, it mainly takes place through an additional entry site, called “cytostome” that represents a round opening at the plasma membrane near the flagellar pocket, which is absent in both *T. brucei* and *Leishmania* (Soares and de Souza, 1991; Porto-Carreiro et al., 2000). The endocytic activity in all these organisms depended on the stage of their life cycle. While *T. brucei* bloodstream form displayed high rates of endocytic activity, this activity was absent or significantly reduced in procyclic form (Morgan et al., 2002a,b). Involvement of TbAct1 during this process has been shown by down regulating TbAct1 expression in blood stream stage of *T. brucei*, using RNAi, and then observing significantly reduced receptor-mediated uptake of transferrin (García-Salcedo et al., 2004). Further, *T. cruzi* epimastigotes possessed high endocytic activity (Bogitsh et al., 1995; Corrêa et al., 2008), whereas in trypomastigotes, a stage that lacks cytostome structure, this activity was low (Soares and de Souza, 1991; Figueiredo et al., 2004). That TcAct1 is involved in this process has been demonstrated in *T. cruzi* epimastigotes by observing inhibition of endocytosis of peroxidase, LDL and gold particles after disrupting actin cytoskeleton by treatment with cytochalasin B and latrunculin B (Soares and de Souza, 1991; Bogitsh et al., 1995; Corrêa et al., 2008). Furthermore, endocytosis was downregulated in *L. mexicana* promastigotes, as compared to both metacyclic promastigotes and amastigotes (Ali et al., 2012). Role of actin during endocytosis in *Leishmania* has been established by observing significantly reduced uptake of the fluorescent dye FM4-64 after inhibiting the LdCof-driven LdAct1 dynamics in *L. donovani* promastigotes (Tammana et al., 2010).

Together with actins, trypanosomatids encode for at least five actin-like proteins (ALPs). Out of which, three proteins, viz. ALP1, ALP3 and ALP4, have been characterized in *T. brucei* and *Leishmania*. These proteins were first identified as a part of the flagellar proteome of *T. brucei and L. mexicana* (Broadhead et al., 2006; Beneke et al., 2019) and thereafter, their localization to the flagellum was confirmed by fluorescent tagging during the genome wide search to assign their location within the *T. brucei* cells (Dean et al., 2017; Halliday et al., 2019). Similar distribution has also been observed earlier by overexpressing fluorescently tagged version of ALP3 in *T. brucei* (Ersfeld and Gull, 2001). However, in *L. donovani*, ALP3 (earlier classified as Arp1) was predominantly localized to the mitochondrion, besides localizing to the cytoplasm and the flagellum (Singh et al., 2014). And depletion of its intracellular levels resulted in decreased mitochondrion membrane potential and the ATP content, and also in shortening of the flagella length. These effects were, however, reversed by episomal complementation of LdALP3 gene (Singh et al., 2014), suggesting that ALP3 regulates mitochondrion potential, ATP synthesis and flagellum length in *Leishmania* promastigotes. The difference observed between intracellular distributions of TbALP3 and LdALP3 may perhaps be attributed to the larger size of LdALP3 (483 amino acids), compared with TbALP3 (433 amino acids), which is perhaps caused by insertions that confer distinctive properties to this protein.

### Actin Binding Proteins (ABPs) in Trypanosomatids

A large number of proteins (>150) bind actin to regulate its functions in higher eukaryotic cells. However, because of their limited functions, lower eukaryotic organisms, such as trypanosomatids, express only a small repertoire of ABPs that are sufficient to meet their requirement. Analysis of genomic data of trypanosomatids revealed that *T. brucei*, *T. cruzi and Leishmania spp.*, encode at least one copy each of profilin, ADF/cofilin, twinfilin, CAP/Srv2 and coronin, whereas variable number of formins and myosins are encoded in these organisms (Table 2). Further, *T. brucei* and *Leishmania spp.* encode two copies each of formins and myosins, while *T. cruzi* encodes many myosins and three formins. Besides this, *T. cruzi* encodes for two copies of CAPz, which is absent in both *T. brucei* and *Leishmania spp*. This apart, all the seven subunits of the Arp2/3 complex (viz. Arp2, Arp3, ARPC1, ARPC2, ARPC3, ARPC4 and ARPC5) are encoded by *T. cruzi* and *T. brucei*, but only four to five subunits of this complex appeared to be encoded in *Leishmani*a *spp.*. Other proteins such as thymosin β4, gelsolin, fimbrin, villin, α-actinin, plastin, spectrin and filamin are completely absent in trypanosomatids. Out of the limited set of ten core ABPs (profilin, twinfilin, ADF/cofilin, CAP/srv2, CAPz, coronin, two myosins, two formins), only a few ABPs have so far been characterized.

#### Actin Filament Nucleating Proteins

Actin polymerization in itself is an energetically unfavorable process till three actin monomers associate together to form a stable nucleus for further polymerization, and this stage is referred to as the “lag phase.” The lag phase is, however, removed *in vivo* due to participation of ABPs, viz. the Arp2/3 complex and formins, which ensure rapid nucleation of actin monomers and thus significantly accelerate actin polymerization. On one hand, the Arp2/3 complex promotes the growth of new filaments by the side of the existing filaments, which is important in dendritic branching found at the leading edge of a lamellipodium of motile cells (Pollard and Borisy, 2003). On other hand, formin proteins promote actin assembly by directing rapid nucleation and elongation of unbranched actin filaments. Besides this, these proteins also assist formation of a variety of actin-structures, including stress fibers, filopodia, and lamellipodia, and modulate the stability and organization of microtubules (Pollard, 2016). This dual activity of formins helps them to coordinate the activities of these two cytoskeleton networks, which allows them to regulate various cellular processes, such as assembly of contractile ring, centrosome assembly, centriole duplication, and centrosome positioning (Breitsprecher and Goode, 2013). The majority of trypanosomatids encode for two formins, but to date, none of these proteins has been characterized.

#### Actin Filament Elongating Proteins

**FIGURE 7:**
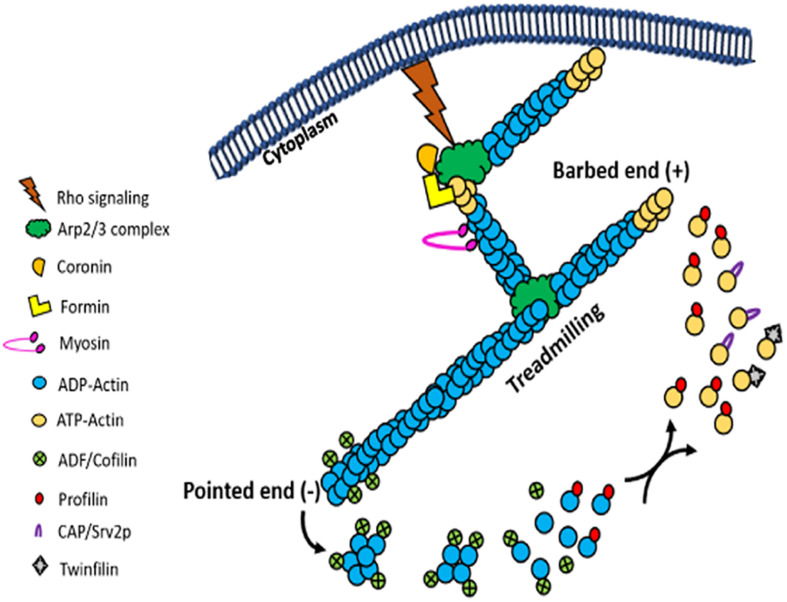
Picture cartoon of actin treadmilling, showing that the rate of treadmilling is regulated by ADF/cofilins and profilin, which results in an increase and decrease in the size of actin filaments, respectively. It further shows that Arp2/3 complex nucleate new filaments by its binding with actin monomers and the side of actin filaments, while formins nucleate new filaments by binding actin monomers and through cooperation of profilin.

After nucleation, actin filaments can grow rapidly upon addition of actin monomers to their barbed ends. Filament length is controlled by capping proteins. While gelsolin and tensin cap the barbed ends of growing actin filaments by blocking addition of new monomers at this end, the pointed end cappers reduce loss of actin monomers from the pointed end and thereby promote rapid extension of the filament. Besides serving as barbed end capper, gelsolin also displays filaments severing activity, which accelerates actin dynamics. The best characterized proteins that drive depolymerization are the actin depolymerizing factor (ADF) and the cofilin family members (ADF/cofilin). ADF/cofilin family of proteins are ubiquitous highly conserved, low molecular-weight ABPs that depolymerize F-actin into actin monomers and consequently increase the turnover of actin filaments (dos Remedios et al., 2003; Ono, 2007). In addition, these proteins exhibit actin filament-severing activity that generates new barbed ends, which accelerates the filament assembly (Ono, 2007). By virtue of their ability to increase the rate of actin turnover at the steady state, ADF/cofilin family of proteins have been implicated in the treadmilling process (Figure 7; Ono, 2007). Although gelsolin and tensin are completely absent in trypanosomatids, one copy of ADF/cofilin is encoded by all these organisms. Amongst these, *T. brucei* and *L. donovani* ADF/ cofilins have been structurally and functionally characterized. *Leishmania* ADF/cofilin (LdCof) bound to both monomeric and filamentous LdAct1 and displayed filament-depolymerizing and severing activities (Tammana et al., 2008; Kumar et al., 2012), whereas *T. brucei* ADF/cofilin (TbCof) bound to only monomeric actin, but similar to LdCof, it possessed filament-depolymerizing and severing activities. Further, both the proteins were co-distributed with actin throughout the cell body, including the flagellum (Tammana et al., 2008; Dai et al., 2013). In addition, both the proteins had similar structures which consisted of a conserved ADF/cofilin fold with a central mixed β-sheet formed of six β-strands, which was surrounded by five α-helices (Pathak et al., 2010; Dai et al., 2013). These proteins possessed conserved G/F-actin binding site that included the characteristic long kinked α-helix (α3).

**FIGURE 8:**
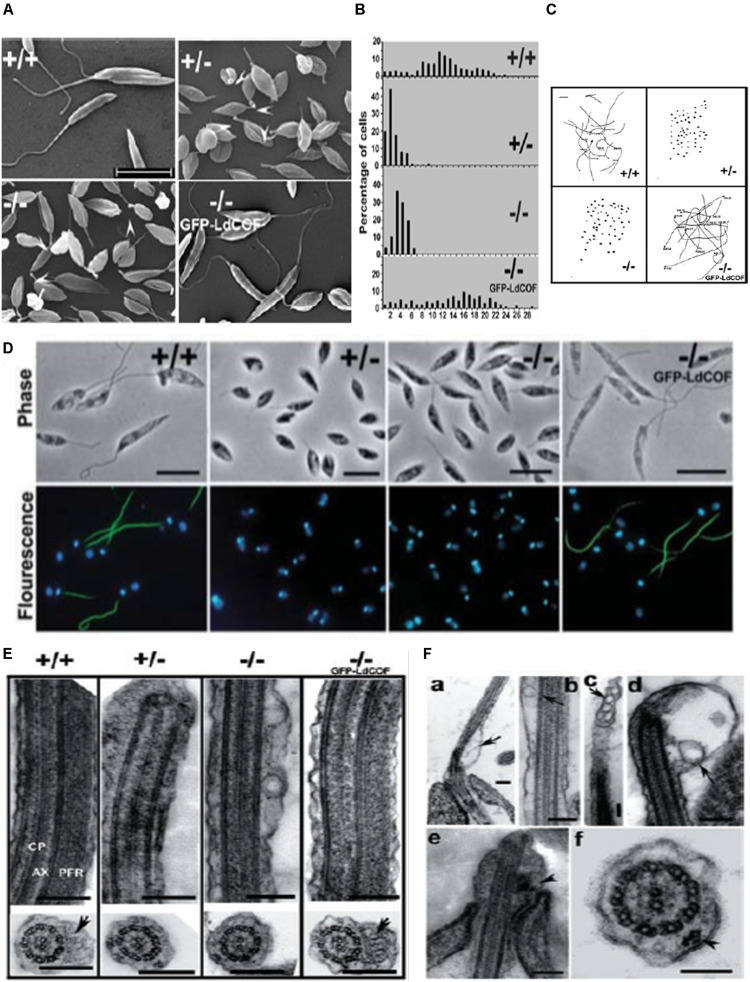
**(A)** Scanning electron micrographs, showing short and stumpy cell body with significantly shortened flagella of heterozygous (+/-) and homozygous (-/-) LdCof mutants, compared with wild type (+/+) cells. Episomal complementation of LdCof^– /–^ cells with LdCof gene (-/- comp) restored the wild-type morphology and flagellar length. Bar, 10 μm. Arrowheads indicate the “blob-like” structures seen at the tip of the flagella of mutant cells (taken from Tammana et al., 2008 with permission). **(B)** Histogram, showing flagellar lengths of LdCof^+/+^, LdCof^+/–^, LdCof^– /–^ and LdCof^–/–comp^ cells (taken from Tammana et al., 2008 with permission). **(C)** Motility analysis of LdCof mutants by time lapse microscopy. Traces of paths of live, individual cells in the movies indicate that LdCof^+/–^ and LdCof^– /–^ cells are completely immotile. However, upon episomal complementation of LdCof^– /–^ cells the motility is restored back to normal. Origin of the path is indicated by solid dots. Bar, 50 μm (taken from Tammana et al., 2008 with permission). **(D)** Immuno -flourescence micrographs, showing loss of paraflagellar rod proteins, PFR1 and PFR2, after staining the LdCof ^+/–^ and LdCof ^– /–^ mutants with mAb2E10 antibodies and their restoration after complimenting LdCof gene in the null mutants. Bar, 5 μm (taken form Tammana et al., 2008 with permission). **(E)** Transmission electron micrographs of thin sections of flagellum from chemically fixed whole cells showing the absence of PFR in LdCof ^+/–^ and LdCof ^– /–^ cells and its restoration upon episomal complementation. Longitudinal sections of the flagellum showing the axoneme (AX) with the central pair microtubules (CP) and PFR confined between the axoneme and the flagellar membrane in wild type cells and GFP–LdCof complemented mutants. Bar, 200 nm. Cross sections of the flagellum, showing the PFR in LdCof ^+/+^ (marked by arrow) and GFP–LdCof-complemented mutant cells, and complete absence of this structure in the cross sections of LdCof^+/–^ and LdCof^– /–^ cells. Bar, 200 nm (taken from Tammana et al., 2008 with permission). **(F) (a–d)** Longitudinal sections of chemically fixed whole cells of LdCof^– /–^ mutants, showing accumulation of membrane-bound vesicles at the base **(a)**, along the length **(b,c)** and tip **(d)** of the flagellum. Arrows indicate the membrane-bound vesicles. Longitudinal section **(e)** and cross section **(f)** of chemically fixed whole cells of LdCof^– /–^ mutant, showing IFT-like particles along the length of the flagellum. Arrowheads indicate IFT-like particles. Bar, for panels **(a–e)** 200 nm and for panel **(f)** 100 nm. IFT, intraflagellar transport (taken from Tammana et al., 2008 with permission).

ADF/cofilin-driven actin dynamics regulates a number of important cellular activities, such as motility, endocytosis, vesicular trafficking, cell division etc. (Pollard and Borisy, 2003). Similar to other eukayotic ADF/cofilins, trypanosomatid ADF/cofilin, especially LdCof, regulates the cell morphology, motility, endocytosis, vesicular trafficking and early phase of cell division in *Leishmania* promastigotes, as revealed by the reverse genetic experiments (Tammana et al., 2008, 2010). The heterozygous and homozygous LdCof mutants prepared through targeted LdCof gene replacement by the selective marker gene, lost not only their motility, but their flagella were completely devoid of the paraflagellar rod (PFR) and length of their flagellum was significantly shortened (Tammana et al., 2008). Additionally, these cells were short and stumpy that contained vesicle-like structures throughout the length of their flagellum (Figure 8). However, all these changes were restored to normal by episomal complementation of the LdCof gene (Tammana et al., 2008, 2010). Further studies are, however, required to evaluate the functions of ADF/cofilin-driven actin dynamics in *Leishmania* amastigotes and in other trypanosomatids.

#### Actin Monomer Binding Proteins

In motile cells, a rapid growth and reorganization of actin filaments, in response to both intracellular and extracellular stimuli, is required, which is dependent on the availability of polymerizable pool of actin monomers. Although there are a large number of actin monomer binding proteins, only six major classes of proteins are found in most eukaryotic organisms (Winder and Ayscough, 2005). The monomer-binding proteins, on one hand, are involved in binding ADP-actin soon after its release from filament ends (e.g., twinfilin, ADF/cofilin), while on the other, they facilitate the exchange of ADP for ATP (e.g., profilin and CAP) and then deliver ATP-bound actin monomer to the barbed ends to facilitate new rounds of polymerization (e.g., twinfilin, Srv2/CAP, profilin, verprolin/WIP and WASP). All trypanosomatids examined to date encode for only four actin monomer binding proteins, viz., profilin, ADF/cofilin, twinfilin and CAP/Srv2, that are involved in actin turnover. Amongst these, LdCof, profilin (LdPfn) and twinfilin (LdTwf) in *L donovani*, TbCof and profilin (TbPfn) in *T. brucei* and only profilin (TcPfn) in *T. cruzi* have so far been characterized.

Profilins are low molecular weight actin monomer binding proteins (Theriot and Mitchison, 1993) that regulate actin dynamics in eukaryotic cells. These proteins are involved in a variety of actin-driven cellular processes, such as motility, vesicular trafficking, chromatin remodeling, nuclear actin export, membrane signaling, etc. (Wilkes and Otto, 2003). Profilins, on one hand, display actin monomer sequestering activity, while on the other, they catalyze nucleotide exchange on actin monomers and also recycle ATP-bound actin monomers to the barbed end (+ end), thereby significantly promote the polymerization process (Witke, 2004; Carlier and Pantaloni, 2007; Krishnan and Moens, 2009). Besides the actin-binding site, profilins also contain two additional binding sites-one for polyphosphoinositides and the other for poly-L-proline (PLP) motives (Sohn and Goldschmidt-Clermont, 1994; Jockusch et al., 2007). The PLP binding domain in profilins is comprised of their N- and C-terminal helices that form PLP binding cleft (Metzler et al., 1994; Mahoney et al., 1999). It is through the PLP binding domain that profilins bind a large number of proteins. While a number of such binding proteins help profilin in regulation of actin dynamics, other proteins partner with profilin in regulating endocytosis, nuclear export, and Rac/Rho effector protein signaling (Witke, 2004; Jockusch et al., 2007). Besides this, binding of profilin to actin (Lassing and Lindberg, 1985) as well as to PLP has been shown to be regulated through its binding to PI (4,5) P2 (Lambrechts et al., 1997).

In trypanosomatid profilins, LdPfn is the most characterized protein (Ambaru et al., 2020). This protein besides localizing to the cytoplasm, it was also localized to the flagellum, the nucleus and the kinetoplast. Under *in vitro* and *in vivo* conditions, LdPfn bound to monomeric actin and *in vitro* it catalyzed nucleotide exchange on G-actin. At its low concentrations, LdPfn promoted actin polymerization, whereas at high concentrations, it strongly inhibited the polymerization process by sequestering actin monomers. This was in accordance with the earlier studies which have shown that in protozoan organisms, such as *Acanthamoeba*, *Chlamydomonas* and *Toxoplasma*, profilins mainly function as actin sequestering proteins (Reichstein and Korn, 1979; Tseng and Pollard, 1982; Kovar et al., 2001; Skillman et al., 2012). Besides actin, LdPfn also bound to PLP motifs and polyphosphoinositides *in vitro*. However, among phosphoinositides, it bound more efficiently to PI (3,5) P2, which is found on early or late endosomes and lysosomes (Wallroth and Haucke, 2018), as compared to PI (4,5) P2 and PI (3,4,5) P3 (Ambaru et al., 2020). Further, LdPfn heterozygous mutants, prepared through targeted replacement of LdPfn gene by selective marker gene, grew at much slower pace, compared to wild type cells, in culture, and displayed slower intracellular trafficking activity (Ambaru et al., 2020). These defects were, however, reversed upon episomal complementation of LdPfn gene, indicating that profilin plays an important role in intracellular trafficking. Furthermore, the slower growth of the heterozygous mutants could perhaps be due to aberrations in the cell division cycle of these cells, which needs to be further explored. Unlike LdPfn, TbPfn and TcPfn have been partially characterized. Expression of TcPfn in different developmental stages of *T. cruzi* (Osorio-Méndez et al., 2016) has been determined, and the protein ligands that might interact with this protein in *T. cruzi* epimastigotes were analyzed by mass spectrometry. TcPfn was expressed in all the developmental stages of the parasite and possibly interacted with a large number of potential ligands, including actin, microtubule components and elongation factor 1α (Osorio-Méndez et al., 2016). However, role of these interactions of TcPfn in cellular functions needs to be determined. Further, profilin expression in *T. brucei* has been demonstrated only at the mRNA level, and the gene encoding for this protein has been shown to complement a yeast mutant lacking profilin (Wilson and Seebeck, 1997). Further studies on these proteins are, however, required to evaluate their biochemical and functional properties.

**FIGURE 9:**
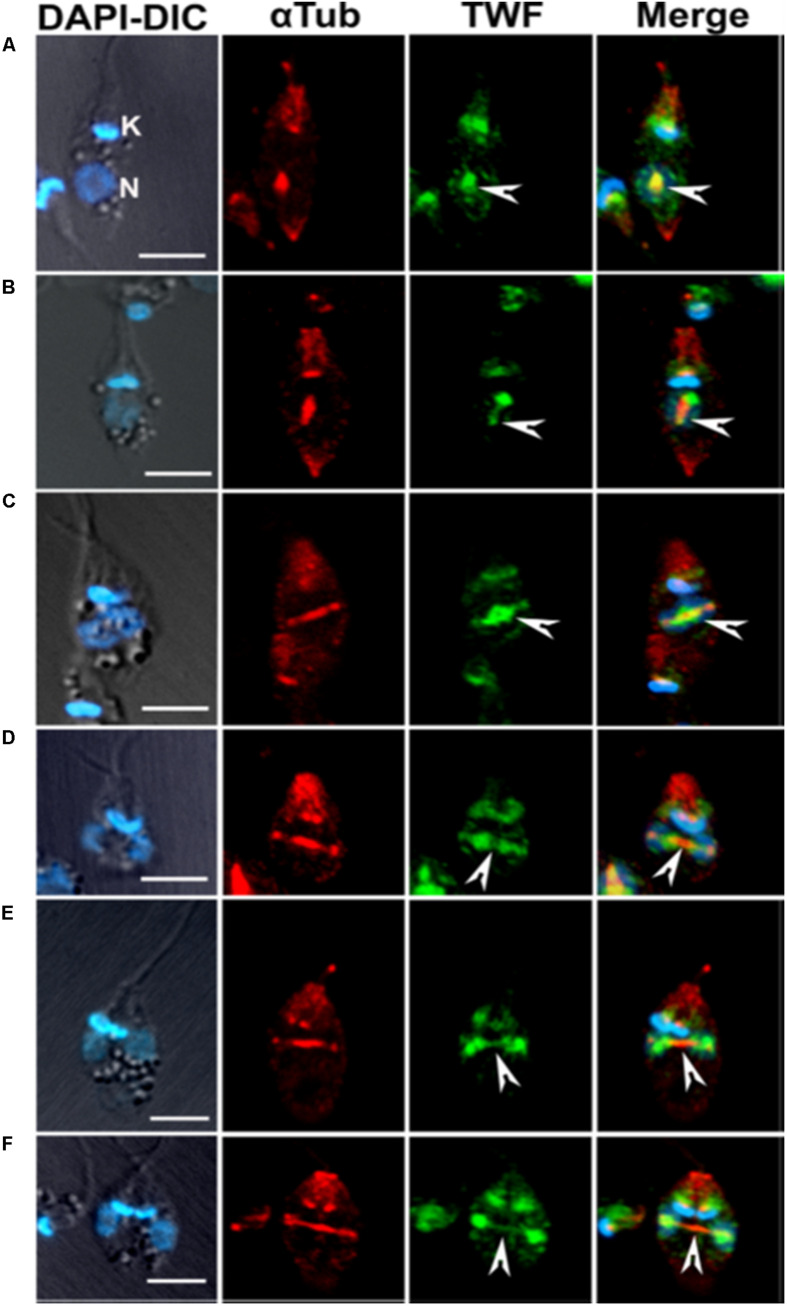
Immunofluorescence micrographs **(A–F)** after staining the cells with anti-LdTwf antibodies, showing movement of twinfilin (Twf) from the nucleolus to origin of the mitotic spindle where it completely localized on the extending spindle microtubules and finally redistributed to the spindle poles. Arrow heads mark distribution patterns of TWF on the spindle, showing the presence of residual TWF on the spindle microtubules while the larger TWF bulk migrated to the poles in the later stages of karyokinesis. Mitotic spindle has been marked by anti α-tubulin (aTub) antibody. Bar, 5 mm (taken from Kumar et al., 2016 with permission).

Another actin monomer binding protein of ADF/cofilin family, twinfilin, has also been characterized in *L. donovani*, but not in other trypanosomatids. *Leishmania* twinfilin (LdTwf), unlike other eukayotic twinfilins (Goode et al., 1998), was mainly localized to the nucleolus and only to a small extent, it distributed in the basal body region in the promastigotes. However, in the dividing cells, it redistributed to the mitotic spindle (Figure 9) and stayed there partly associated with the spindle microtubules (Kumar et al., 2016). In addition, the growth of heterozygous LdTwf mutants, prepared by targeted LdTwf gene replacement by the selective marker gene, was considerably decreased due the delayed nuclear DNA synthesis and altered mitotic spindle length and architecture, suggesting that twinfilin harmonizes karyokinesis in *Leishmania* promastigotes (Kumar et al., 2016). Although all twinfilins characterized to date have been shown to interact with monomeric actin, no such interaction of LdTwf with LdAct1 could be demonstrated *in vivo* or *in vitro* in this study (Kumar et al., 2016), suggesting that LdTwf function in the nucleus could be independent of LdAct1. The other class of proteins that make a free pool of actin monomers available in motile cells is of actin sequestering proteins, such as the thymosin family of proteins. These proteins act by clamping ATP actin top to bottom, to effectively cap at both barbed and pointed ends and thus prevent its incorporation into filaments (Hertzog et al., 2004; Irobi et al., 2004). Appropriate signals at the cell cortex can then trigger activation of profilin, which results in a rapid release of thymosin binding, leading to a large increase in the polymerizable pool of free ATP-actin. However, this family of proteins are not encoded by the trypanosomatids genomes.

#### Actin Filament Bundling Proteins

There are other ABPs that participate in filament bundling (e.g., fimbrin, coronin), filament crosslinking (fimbrin, α-actinin, and filamin) and filament stabilization (e.g., tropomyosin, troponin), but out of these proteins, trypanosomatids genomes encode only for coronin. Coronins are F-actin binding proteins present in most eukaryotic cells, except the plant cells (Xavier et al., 2008; Chan et al., 2011), that play important role in numerous cellular functions, such as cell motility, phagocytosis, cytokinesis, etc. (Combaluzier and Pieters, 2009; Ishikawa-Ankerhold et al., 2010; Shina et al., 2011; Xavier et al., 2012; Tchang et al., 2013). Together with other ABPs, such as Arp2/3 complex, cofilin and actin-interacting protein-1, coronins are known to participate in reorganization of actin-network (Goode et al., 1999; Humphries et al., 2002; Kueh et al., 2008; Gandhi et al., 2009; Lin et al., 2010; Xavier et al., 2012; Jansen et al., 2015). Of the trypanosomatid coronins, only *Leishmania* coronin (LdCor) has been characterized. This protein colocalized with LdAct1 filaments and its overexpression promoted filament formation in *Leishmania* promastigotes (Nayak et al., 2005).

The most characteristic structural feature of all coronins is that they contain five WD repeats, a leucine zipper motif and a coiled-coil domain at their C-terminus (de Hostos, 1999). All these structural features were fully conserved in LdCor structure, except that its leucine zipper motif contained as many as five heptads, rather than 2–3 heptads found in this structural region of other coronins (Nayak et al., 2005). As the number of heptads in the leucine zipper motif determines the degree of coronin oligomerization wherein the coiled-coil domains play a significant role (Asano et al., 2001: Oku et al., 2005), LdCor, unlike other coronins which mainly exist as dimers or trimers, formed higher order oligomers (tetramer or pentamer) through its coiled-coil domain (Srivastava et al., 2015). This was confirmed by determining the 3-d structure of the LdCor coiled coil domain by X-ray crystallography (Nayak et al., 2016). Results revealed an anti-parallel tetramer assembly of the coiled coil domain. Further analysis using small angle X-ray scattering and chemical crosslinking confirmed the existence of tetrameric form of this domain in solution, which was consistent with the observed oligomerization of full-length LdCor (Nayak et al., 2016). In addition, truncation of the coiled-coil domain ablated the ability of LdCor to assist LdAct1 filaments formation, suggesting that the coiled-coil domain was essential only for LdCor oligomerization but not for interaction of LdCor with LdAct1 filaments (Srivastava et al., 2015). Instead, LdCor unlike other coronins, interacted with actin-filaments through its unique region (Srivastava et al., 2015). Besides this, LdCor preferentially distributed to the distal tip during cytokinesis in *Leishmania* cells, where it interacted with the microtubules through a microtubule-based motor, kinesin K39 (Sahasrabuddhe et al., 2009). And in LdCor depleted (by about 50%) dividing cells, about 25–30% log phase cells possessed bipolar morphology, which was primarily due to an uncoordinated growth of the corset microtubules (Sahasrabuddhe et al., 2009). Detailed analysis of these cells revealed that the underlying cause of this change in cell morphology was the intrusion of the persistently growing corset microtubules into the other daughter cell corset from the opposite direction. However, the cell morphology was restored to normal by LdCor gene complementation in the LdCor depleted cells, suggesting that coronin regulates the microtubule remodeling during *Leishmania* cytokinesis (Sahasrabuddhe et al., 2009). Although the contribution of LdAct1 during the above process has not been defined in this study (Sahasrabuddhe et al., 2009), it is likely that coronin acts as a link between the actin network and microtubules in trypanosomatids, especially *Leishmania.*

#### Actin-Based Motor Proteins

Myosins constitute a group of proteins that display actin-dependent motor activity and regulate a wide range of functions in eukaryotic cells (Woolner and Bement, 2009). These proteins are comprised of a conserved N-terminal motor domain, a neck region including the IQ motifs for calmodulin (light chain) binding, and a C-terminal cargo-binding tail domain that confers functional specificity on different classes of myosins (Krendel and Mooseker, 2005). While the motor domain is primarily responsible for binding to filamentous actin and hydrolysis of ATP, the tail domain determines its functions in the cells, by controlling the state of oligomerization and selection of specific cargo for transport. Because of the high degree of sequence conservation in the head domain, myosins have been expected to power their movements along F-actin tracks, and divergent tail domain is responsible for binding to a variety of proteins as well as membranes (Karcher et al., 2002). Depending on the domain composition and variations in the amino-acid sequence, myosins have been classified into more than 30 classes in different organisms (Foth et al., 2006; Odronitz and Kollmar, 2007)). Trypanosomatid family of organisms encode for two myosins: myosin 1 (Myo1), and a kinetoplastid-specific class XXI myosin, which after phylogenetic analysis of trypanosomatid myosins, has now been reclassified as myosin13 (de Souza et al., 2018). In addition to these two myosins, *T*. *cruzi* contains additional seven more myosins, which were initially considered “orphans” but recently, in reclassification of trypanosomatids-specific myosins, this group of myosins has been classified into a new class, XXXVI, which included Myo A, Myo B, Myo C, Myo D, Myo E, Myo F, and Myo G (de Souza et al., 2018).

In trpanosomatids, Myo1 in *T. brucei* (TbMyo1), Myo F in *T. cruzi* (TcMyo F) and Myo13 in *L. donovani* (LdMyo13) have been functionally characterized. TbMyo1 was equally expressed in both blood stream and procyclic forms of *T. brucei*, but its distribution differed depending on the parasite life cycle (Spitznagel et al., 2010). In blood stream forms of *T. brucei*, TbMyo1 localized to the polarized endocytic pathway in TbAct1 dependent manner (Spitznagel et al., 2010), and its knock down by RNAi resulted in a significantly reduced endocytic activity, flagellar pocket enlargement, termination of cell division and finally cell death (Spitznagel et al., 2010). In contrast, no such changes in growth or morphology were observed even after loss of 90% of TbMyo1 in procyclic forms, suggesting a life cycle stage specific requirement for TbMyo1 in endocytosis and cell division in *T. brucei* (Spitznagel et al., 2010). In *T. cruzi*, the orphan myosin, Myo F, has recently been identified as the enzymatic component of the cytostome-cytopharynx complex that this parasite utilizes for endocytosis (Chasen et al., 2020). The dominant negative mutants prepared by overexpression of TcMyo F, although did not lose their viability, were shown to be completely deficient in endocytic activity. However, full deletion of TcMyo F gene resulted only in a decrease in the rate of endocytosis, potentially indicating toward the role of other myosins in the endocytic process (Chasen et al., 2020). Further analysis revealed involvement of three additional orphan myosins, two of which (Myo B and Myo E) were targeted to the preoral ridge region adjacent to the cytostome entrance and the other (Myo C) was targeted to the cytopharynx tubular structure similar to Myo F (Chasen et al., 2020). It was proposed that while the myosin motors targeted to the preoral ridge region (Myo B and Myo E) could function to move bound surface cargo to the cytostome, those myosins on the tubular cytopharynx (Myo F and Myo C) may then transport endocytosed vesicles to the posterior reservosomes (Chasen et al., 2020).

*L. donovani* encodes for two myosins, viz. Myo1B and Myo13, of which LdMyo13 is a trypanosomatid-specific myosin that contains two ubiquitin associated (UBA)-like domains toward the end of its C-terminus. LdMyo13 is expressed in both the stages of *Leishmania* life cycle, viz. promastigote and amastigote stages. However, its expression in the amastigote stage was about 20 times reduced, compared with the promastigote stage (Katta et al., 2009). In the promastigotes, LdMyo13 besides localizing to the cytoplasm was also prominently localized at the base of the flagellum, where it appeared to partly associate with the PFR (Katta et al., 2009). Further studies revealed that the flagellar localization was exclusively determined by the LdMyo13 tail region wherein UBA- like domains played a crucial role (Katta et al., 2009; Bajaj et al., 2020)). Besides this, expression of LdMyo13 varied during growth of *Leishmania* cells in culture with greater expression at the stationary phase, compared with the early or mid-log phase (Katta et al., 2010). Further, detergent treatment of the promastigotes gave rise to two fractions of LdMyo13- detergent-soluble and detergent-insoluble (Katta et al., 2009), indicating existence of two populations of LdMyo13 in the flagellum of which one population was associated with the flagellar cytoskeleton, while the other population perhaps served as an actin-dependent motor. This apart, similar to LdCof (*cf* Figure 8), depletion of the LdMyo13 intracellular levels by about 50% resulted in loss of PFR and cell motility, significantly reduced flagellar length, enlargement of the flagellar pocket and impairment of the intracellular trafficking (Katta et al., 2010). These defects were, however, reversed by episomal complementation of LdMyo13 gene in the LdMyo13-depleted cells.

Analysis of LdMyo13 amino acid sequence revealed the presence of an N-terminal motor domain, a neck region including IQ motives for light chain (calmodulin family of proteins) binding, and a C-terminal cargo-binding domain. The end of the motor domain contained a coiled coil region with a strong tendency to dimerize. This region partially overlapped with the PX domain, which has been shown to bind anionic phospholipids (Batters et al., 2014). It has been further reported that the tail domain contained as many as six nonspecific binding sites for lipids of which two such sites overlapped with the region (aa953 – aa1050) where two UBA domains were located in the LdMyo13 sequence (Batters et al., 2014). Depending on the presence/ binding of calmodulin, LdMyo13 adopted monomeric or dimeric states *in vitro* (Batters et al., 2012). While binding of LdMyo13 to single calmodulin was shown to produce a monomeric state with an ability to move actin filaments (Batters et al., 2012), without calmodulin binding, only non-motile dimers were formed that crosslinked actin filaments (Batters et al., 2014), suggesting that LdMyo13 could exist in both the monomeric and dimeric states. This was consistent with the presence of two populations of LdMyo13 in *Leishmania* promastigotes (Katta et al., 2009). Further, only the LdMyo13 monomers but not the dimers, could bind lipids, suggesting that calmodulin-bound LdMyo13 may transport lipid cargos during assembly and disassembly of the promastigote flagellum. In addition, *in vitro* studies, using pure proteins, revealed that LdMyo13 binds along the length of actin filament ends, and that calmodulin binding was essential for actin filaments translocation (Batters et al., 2012).

## Discussion

It is evident from the preceding sections that only a limited information is available on the structure and functions of trypanosomatid actins and ABPs. Despite their belonging to the same family, all the three organisms, viz. *T. brucei*, *T. cruzi* and *Leishmania spp*., encode differing number of actins, actin binding, actin-like and actin related proteins, indicating a complex regulation of actin cytoskeleton in these organisms. Amongst these, *T. cruzi* actin cytoskeleton seems to be the most complex, as unlike *T. brucei* and *Leishmania spp*., this organism encodes for four actins, many ABPs and several myosins, all of which belong to the novel class of myosins. This clearly calls for more concerted efforts to decipher the structural and functional features of these proteins.

Unlike conventional actins, trypanosomatid actins mainly exist in form of granules, patches and bundles rather than thin filaments having 7–10 nm thickness. As given in Section “Brief Overview of Conventional Actins,” during filament formation, subdomains 1 and 3 of one actin monomer associate with subdomains 2 and 4 of another monomer. A part of amino acid sequences that are contributed by subdomain 2 in this process constitute the DNaseI binding site, including an eleven aa residues loop which stabilizes the filament structure. As this loop in LdAct1 is highly diverged, compared to conventional actins (Sahasrabuddhe et al., 2004; Kapoor et al., 2008), it may lead to destabilization of the filament structure by affecting the monomer-monomer associations within the filament. This could perhaps be the reason for inability of trypanosomatid actins to form stable filaments. Further, most of the diverged aa residues in LdAct1 are exposed on its surface, which may result in altered surface topology and consequently in altered monomer-monomer associations that may mask the phalloidin binding sites in LdAct1oligomeric structures (Kapoor et al., 2008).

It is apparent that some of the functions of trypanosomatid actins, such as regulation of endocytic and intracellular trafficking activities, appear similar to that of canonical (or conventional) actins. However, despite having more than 90% sequence identity to TbAct1 and TcAct1, LdAct1 unlike TbAct1 and TcAct1, localized to the nucleus and kinetoplast and surprisingly displayed *in vitro* supercoiled DNA relaxing and kDNA nicking activities (Sahasrabuddhe et al., 2004; Kapoor et al., 2010), which might have been required during the chromatin and kDNA remodeling in *Leishmania* spp. This suggests that functional diversity of trypanosomatid actins and ABPs is determined by the functional requirements of the specific organism. Further, despite their structural diversity, trypanosomatid myosins, viz. TbMyo1, TcMyo F and LdMyo13, similar to canonical myosins, function as actin dependent motors in regulating endocytosis and intracellular trafficking in the respective organisms. Intriguingly, unlike *T. brucei* and *Leishmania spp*., where single isoform of myosin is sufficient to accomplish motor functions during endocytosis and intracellular trafficking, in *T. cruzi* as many as four myosins, viz. TcMyo B, TcMyo C, TcMyo E and TcMyo F, appear to be required to accomplish the same functions (Chasen et al., 2020). This aspect of regiospecific function of *T. cruzi* myosins is quite fascinating and needs to be further explored in detail.

The scDNA-relaxing and k-DNA nicking activities of LdAct1 together with its presence in the nucleus and the kinetoplast (Sahasrabuddhe et al., 2004; Kapoor et al., 2010) indicate that this protein perhaps plays some important role in these organelles. Actin in eukaryotic cells has been shown to be involved in several nuclear processes, such as chromatin remodeling, DNA repair and regulation of transcription (Bettinger et al., 2004; Miralles and Visa, 2006; Percipalle and Visa, 2006; Chen and Shen, 2007; Hurst et al., 2019). The SWI/SNF and INO80 families of chromatin remodeling complexes contain actin and Arps as their subunits that bind directly to each other (Olave et al., 2002; Kapoor and Shen, 2014). So far, only three Arps have been analyzed in *Leishmania*, out of which LdArp2 and LdArp3 were exclusively localized to the cytoplasm, whereas over-expressed version of LdArp6 was localized to the nucleus (Raza et al., 2007). As Arp6 is an essential component of the SRCAP/SWR1 chromatin remodeling complex, which deposits the histone variant H2A.Z into chromatin (Oma and Harata, 2011), the possible association of Arp6 with LdAct1 has been analyzed by the ChIP assay, using anti-LdArp6 antibodies (Raza et al., 2007). Analysis of the immunoprecipitated chromatin revealed the absence of actin in the precipitated material, suggesting that LdAct1 is not a component of the SRCAP complex. Further studies are required to ascertain the functions of LdAct1 in the *Leishmania* nucleus.

Trypanosomatids, unlike other eukaryotic cells, contain highly complex network of mitochondrial DNA which is exclusively localized to a fixed region of the mitochondrial matrix, near the basal body region, called “kinetoplast.” The kinetoplast DNA (kDNA) is a network of circular DNAs containing two types of DNA circles, viz. minicircles and maxicircles. Depending on the species, kDNA contains 5,000–10,000 minicircles (0.5–10 kb in length) and 25–50 maxicircles (20–40 kb in length), which are catenated to form a highly condensed disk-like structure (Chen et al., 1995; Klingbeil et al., 2001; Lukes et al., 2002). In the non-replicating kDNA state, each minicircle is catenated to three neighboring minicircles (three valence state), which are individually covalently closed. However, during replication, the minicircles are in the open state, and consequently the valence state increases with the progression of replication process from three to six, due to constraints imposed by the space available to the network. Nevertheless, as cell proceeds through the growth phase, the valence state again drops back to three due to increase in the space (Lukes et al., 2002). The factors that constrain the network volume in the kinetoplast matrix during replication process still remain unknown, however, a role of mitochondrial membrane or some unknown cytoskeleton structure has been contemplated (Liu et al., 2005). As kinetoplast associated LdAct1 may act as a kDNA nicking enzyme and its filamentous form could provide the required matrix during kDNA replication process, it may be envisaged that LdAct1 could be involved in this process. Nevertheless, it needs to be further confirmed *in vivo* or *ex-vivo* using isolated mitochondria.

**FIGURE 10:**
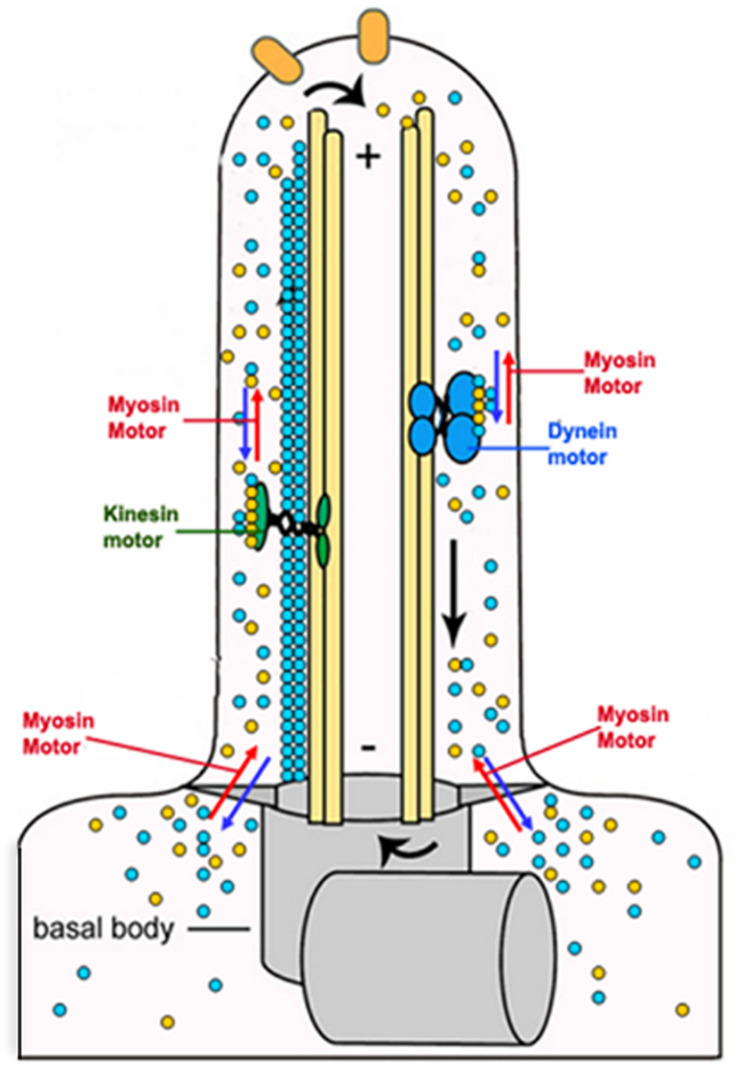
Cartoon diagram showing involvement of the actin based LdMyo13 motor protein in assembly/disassembly of the paraflagellar road (PFR) and the flagellar membrane during remodeling of the *Leishmania* flagellum.

The flagellum is a complex microtubule-based dynamic structure that performs functions related to motility, cell signaling and cell morphogenesis (Hill, 2003; Kohl et al., 2003). Unlike other flagellated organisms that contain axoneme as the sole component of their flagellum, the trypanosomatid flagellum contains an extra-axonemal rod-like structure, called paraflagellar rod (PFR). The flagellum dynamics involves a process of its assembly and disassembly, which requires two-way movements of protein cargoes within the flagellum (Rosenbaum and Witman, 2002). These functions are mainly carried out by the microtubule-based motor proteins, such as kinesin II and dynein complexes (Cole and Snell, 2009). However, a role of actin-dependent LdMyo13 motor has also been speculated in the process (Katta et al., 2010). This was primarily based on the facts that: (I) partial or complete depletion of the intracellular pool of LdCof resulted in loss of PFR and also adversely affected the cell motility, flagellar pocket structure and intracellular trafficking (Tammana et al., 2008), and that (II) similar results were observed also by depleting (∼50%) the intracellular pool of LdMyo13 (Katta et al., 2010). In continuation of these studies, it has recently been shown that the two UBA-like domains located toward the end of the C-terminus of LdMyo13 are essentially required for involvement of LdMyo13 in the flagellum assembly/disassembly process (Bajaj et al., 2020). Further, it has earlier been shown that the proteins released during the flagellum disassembly are transported back to the cytoplasm for their degradation (Maga et al., 1999; Adhiambo et al., 2005), and that during disassembly of the *Chlamydomonas* flagellum, at least 20 proteins get polyubiquitinated prior to their transport and degradation in the cytoplasm (Wang et al., 2019). As UBA-like domains containing proteins bind with polyubiquitinated substrates that are marked for degradation and also with subunits of the proteasome (Su and Lau, 2009), it may be concluded that during the *Leishmania* flagellum disassembly, LdMyo13 may shuttle the released proteins after their presumed ubiquitylation, for degradation through the ubiquitin-proteasome pathway (Heinen et al., 2011). Besides this, as LdMyo13 tail region contains six lipid binding sites that nonspecifically bind anionic phospholipids (Batters et al., 2014), it may be further speculated that LdMyo13 could serve as a lipid transporter during *Leishmania* flagellum assembly and disassembly (Figure 10).

Following the reverse genetic approach, depletion of the intracellular levels of LdALP3 resulted in decreased mitochondrial membrane potential and the ATP content together with shortening of the flagellum length (Singh et al., 2014). Similar shortening of the flagellum has also been observed earlier in case of LdCof and LdMyo13 heterozygous mutants (Tammana et al., 2008; Katta et al., 2010). Although morphologies of the LdCof and LdMyo13 mutants closely resembled the nonmotile clones of LdALP3 mutants, there were two distinct features that made them different from each other – (1) the flexible and fast wriggling flagellum of the LdALP3 mutants, which in case of LdCof and LdMyo13 mutants was completely immotile (Tammana et al., 2008; Katta et al., 2010), and (2) the assembly of PFR (though poor) in LdALP3 mutants, which was absent in the LdCof and LdMyo13 mutants. Based on these facts, it has been concluded that LdALP3 perhaps operates through a mechanism, that appears to be different from the mechanisms through which actin-based LdMyo13 motor functions in assembly of the *Leishmania* flagellum (Singh et al., 2014). However, a cross-talk between the actin-based LdMyo13 motor and LdALP3 operated mechanisms cannot be ruled out.

**FIGURE 11:**
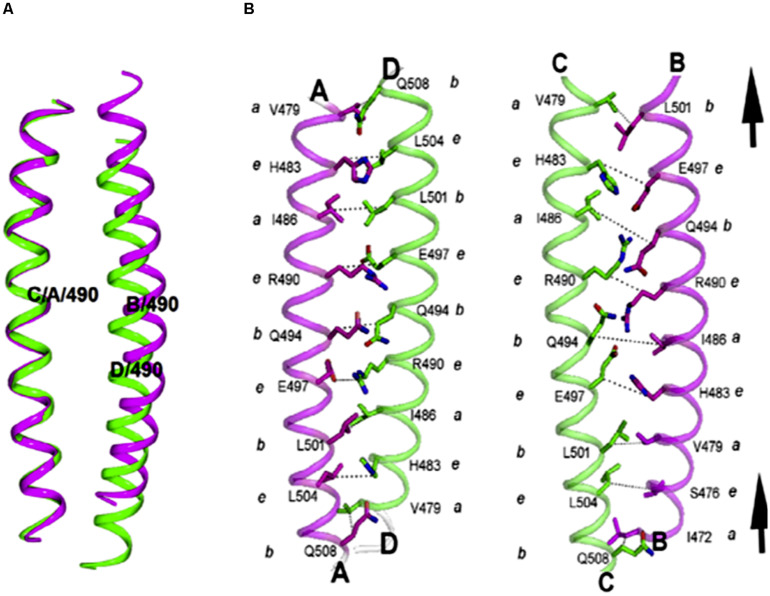
**(A)** Asymmetry observed in the LdCor coiled coil domain tetramer. Cartoon represents the pairs of dimers, highlighting the asymmetry. In left panel, Cα atoms of aa residues 475–507 of chain C were superposed to corresponding atoms of chain A using Superpose (Krissinel and Henrick, 2004) and the transformation applied to the BC dimer. The B and D helices of AD/BC dimers superpose with an RMSD of 3.4 Å. **(B)** Interactions at the BC dimer are different from that of AD dimer due to an upward shift in B helix by a heptad. Also, the distances across the interface are longer in the BC helical interface (Taken from Nayak et al., 2016 with permission).

Trypanosomatid coronins, such as LdCor, display unique structural features, which have not been observed earlier in any other eukaryotic coronin. Most eukaryotic coronins contain the RhXXhE trimerization motif in their coiled coil domain (CC), however, in kinetoplastid coronins the positions of R and E are interchanged within LdCoro CC. Surprisingly, this change in motif affected the oligomeric specificity, which in turn resulted in anti-parallel tetramer assembly rather than the trimer assembly, as revealed by the X-ray crystal structure of the LdCoro CC (Nayak et al., 2016). Interestingly, it also showed that LdCor CC has an inherent asymmetry (Figure 11), in that one of the helices of the bundle was axially shifted with respect to the other three (Nayak et al., 2016). Besides coronin, trypanosomatid twinfilins, especially LdTwf, exhibited novel functional feature in that unlike other eukaryotic twinfilins, it did not bind to LdAct1. Instead, it appeared to partly bind to spindle microtubules, specifically during mitosis (Kumar et al., 2016). However, nothing is known about the structural features of trypanosomatid twinfilins that impart these novel functional features to this class of actin monomer binding proteins.

TbAct1 and TbMyo1 are essential for survival of the blood stream form of *T. brucei*, as knockdown of TbAct1 or TbMyo1 gene by RNAi eventually resulted in cell death (García-Salcedo et al., 2004; Spitznagel et al., 2010). Further, despite repeated attempts, homozygous mutants of LdPfn could not be generated (Ambaru et al., 2020), indicating that this protein is perhaps essential for survival of *Leishmani*a. This is in accordance with the earlier observations that profilin depletion affected the survival of both procyclic and bloodstream forms of *T. brucei* (Alsford et al., 2011). Besides, all attempts to obtain null mutants of LdMyo13, LdCor and LdTwf resulted in changes in ploidy that enabled the parasite to keep back alleles of the wild-type locus (Sahasrabuddhe et al., 2009; Katta et al., 2010; Kumar et al., 2016), and also the drug resistance markers, which frequently occurs in case of *Leishmania* essential genes (Jones et al., 2018). In such cases, appropriate methods are required to generate null mutants of these proteins so as to fully reveal their functions.

LdCof null mutants although reported to have decreased endocytic and intracellular trafficking activities with immotile and paralyzed flagellum, yet these cells grew happily in culture (Tammana et al., 2008, 2010), indicating that LdCof-driven LdAct1 dynamics may not be essential for survival of *Leishmania* promastigotes. However, as LdAct1 and LdMyo13 appear to be essential for survival of the *Leishmania* cells and LdMyo13 motor function is dependent on actin dynamics (Katta et al., 2010), it may be envisaged that some other unknown ABP might have been functioning as actin dynamics regulator in these cells. The actin filament severing function through which ADF/cofilin family of proteins accelerate the actin treadmilling, may be substituted by another ABP, gelsolin (dos Remedios et al., 2003), but this protein is not encoded by the trypanosomatid organisms. Further, from the limited repertoire of ten ABPs that are encoded by these organisms, the actin monomer binding protein, twinfilin, does not behave like other twinfilins in that it failed to bind to LdAct1 and mostly localized to the nucleolus (Kumar et al., 2016). It is, therefore, most likely that there may be other unknown ABPs existing in these organisms that could substitute canonical ABPs functions. Further efforts are required to search for such proteins in trypanosomatid genomes. Besides, detailed functional studies of all the actin-related and actin-like proteins may be undertaken to analyze whether some of these proteins associate with actin to regulate its functions or can themselves function as a substitute of actin.

## Author Contributions

CG conceptualized, wrote, and edited the manuscript. BA prepared the tables, figures, and figure legends. RB collected the literature. BA and RB prepared the first draft. All authors contributed to the article and approved the submitted version.

## Conflict of Interest

The authors declare that the research was conducted in the absence of any commercial or financial relationships that could be construed as a potential conflict of interest.
